# Expiration analysis of the International Space Station formulary for exploration mission planning

**DOI:** 10.1038/s41526-024-00414-3

**Published:** 2024-07-23

**Authors:** Thomas E. Diaz, Emma C. Ives, Diana I. Lazare, Daniel M. Buckland

**Affiliations:** 1 University of North Carolina at Chapel Hill Eshelman School of Pharmacy, Chapel Hill, NC USA; 2 The Johns Hopkins Hospital, Baltimore, MD USA; 3 University of Texas at El Paso School of Pharmacy, El Paso, TX USA; 4 Department of Emergency Medicine, Duke University, Durham, NC USA

**Keywords:** Occupational health, Outcomes research, Pharmacodynamics

## Abstract

Effective medications will be required to maintain human health for long-duration space operations. Previous studies have explored the stability and potency of several of the medications used on the International Space Station (ISS). This study is a comprehensive analysis of the expected terrestrial shelf-lives of the entire 2023 ISS formulary using 4 international registries. Of the 106 medications in the ISS formulary, shelf-life data was found in at least 1 of the registries for 91 (86%) medications. Of these 91 medications, 54 have an estimated terrestrial shelf-life of ≤36 months when stored in their original packaging. 14 will expire in less than 24 months. The results of this study provide operational insight to supplying a pharmacy for an exploration mission, optimize therapeutic outcomes, and prevent diseases associated with extended spaceflight operations. Ultimately, those responsible for the health of spaceflight crews will have to find ways to extend the expiration of medications to the complete mission duration or accept the elevated risk associated with administration of an expired medication.

## Introduction

Space is an extreme environment containing unique stressors which present hazards to human health^1^. In addition, humans who live and work in the space environment are separated from the standard of care available on Earth^2,3^. As the National Aeronautics and Space Administration (NASA) and its international partners plan for long duration, deep space missions, humans will be at a greater risk of adverse medical conditions compared to missions in low earth orbit (LEO)^2^. The use of select pharmacological agents will be one of many countermeasures to mitigate these risks^4,5^.

Astronauts report daily use of medications from medication kits aboard the International Space Station (ISS)^6^. A 2015 retrospective study, which examined medication use during space flights greater than 30 days, discovered pain, congestion, circadian disruptions, and allergies were some of the most common conditions astronauts routinely self-treated aboard the ISS^6^. In addition, a follow-up study utilizing a software-based medication tracker found on average 20.6 ± 8.4 doses of various medications administered per subject per week in flight^7^. However, the expiration of medications, lack of resupply capability, and absence of emergency evacuation present an operational challenge to the medical capabilities during deep space missions, such as a 36 month expedition to and from Mars^8–10^. Therefore, it is imperative to consider the usefulness of a safe and effective pharmacy aboard future spacecraft. Medication stability and potency are a few of the primary factors to consider for supplying a self-sufficient *astropharmacy*, capable of performing Earth Independent Medical Operations (EIMO)^9,11^.

Drug stability evaluation guidance is issued by the International Conference on Harmonisation (ICH) and adopted by the US Food and Drug Administration (FDA) to ensure the quality of a drug substance or product under a variety of factors including time and environmental factors (i.e. temperature, humidity, and light)^12–15^. Stability testing establishes storage conditions as well as a drug retest timelines for drug substances^12,15^. If a drug becomes unstable, its physical or chemical properties may be altered, such as its appearance, potency, dissolution, solubility, or presence of degradation products^13,14^. Manufacturers must perform these tests on at least three primary batches of a drug substance^12,15^. In addition, testing must also be done on a drug substance in the container system it is to be stored and distributed in, as well as under its storage conditions^12,16^. As of 2018, the ICH establishes the acceptable range of drug potency for medications approved by the FDA must fall within 95-105%^12^. Any variation from this range may suggest potential danger to the patient due to lack of efficacy or the presence of toxic degradation products^17,18^. Stability studies of a drug’s active pharmaceutical ingredient (API) guide the medication’s estimated shelf-life^15,16^. Using the estimated shelf-life, manufacturers extrapolate potency data from their stability studies to identify a supported shelf-life and a maximum shelf-life, which are calculated using statistical methods and an ICH decision tree, respectively^15^. The labeled shelf-life, which is printed on the drug product’s label, is defined as the shorter of the supported shelf-life and maximum shelf-life^15^. The labeled shelf-life is then used to calculate the actual expiration date on each drug product^15^. However, it is important to note that this expiry date is the minimum period of time which the drug will remain stable under its sealed packaging in recommended storage conditions. Therefore, it is not a reflection of the drug’s true shelf-life, which may be significantly longer than proposed.

Medications contained in the United States (US) portion of the ISS, as part of the ISS medication kits, are stored and packaged in the US prior to flight. In the US, terrestrial shelf-life data are largely unavailable to the public^9,19^. This is primarily because US law does not require expiration dates, use-by dates, or re-test dates on their product labels. Without accurate shelf-lives, humans in long duration, deep-space operations will be at risk of therapeutic failure or toxicity if given a medication past its expiration date^9,11,20^. Ultimately, this gap in knowledge proves the necessity to establish a repository characterizing the labeled shelf-lives of all medications that are to be transported to space.

Through a Freedom of Information Act (FOIA) request, we received a comprehensive manual from NASA containing the medical protocols, procedures, and supplies existing aboard the ISS (received on August 2nd, 2023). This document contains the list of chosen medications and medical equipment stored on the ISS, referred to here as the ISS “formulary.” Each medication within the formulary is divided and assigned to a medication pack based on use. This formulary contains the drug product, strength/volume, route of administration, quantity in pack, unit, possible side effects, comments, and location; however, it does not cite any expiry data. In this study, the term “medications” refers to the products that exist in the ISS formulary, regardless of whether the product contains an actual API (i.e, sterile water). This investigation aims to evaluate the proposed terrestrial shelf-lives of medications stored on board the ISS utilizing publicly available shelf-life data and to identify a shelf-life for each medication, if available.

## Results

As of 2023, there are 111 total medications on board the ISS, split between five different medical kits. Each medical kit is color coded and holds medications pertinent to their designated use. The medication breakdown is as follows: the convenience medication pack holds 23 medications; the emergency medical treatment pack holds 4 medications; the oral medication pack holds 36 medications; the topical & injectable medication pack holds 37 medications; the vascular contingency medication pack holds 11 medications. Two medications exist as a diluent for another medication and were excluded: sterile water for injection and sterile water for reconstitution. Three medications of the same strength and dosage form exist in more than one medication pack, therefore these duplicates were excluded: fexofenadine (Allegra) 180 mg tablets, modafinil (Provigil) 200 mg tablets, and bacitracin 500 units per gram ointment. Consequently, 106 unique drug products were analyzed. No expiration data could be obtained for 14% (*n* = 15) of the ISS formulary. Terrestrial expiration data were obtained for 86% (*n* = 91) of the ISS formulary. Using each medication’s maximum labeled shelf-life, 59.3% (*n* = 54) of the 91 unique medications with expiry data had a terrestrial shelf-life ≤36 months when stored in their original packaging. Using each medication’s minimum labeled shelf-life, 97.8% (*n* = 89) have a terrestrial shelf-life ≤36 months when stored in their original packaging.

Table 1 is the convenience medication pack. There are four medications in it with no publicly available expiry data: bacitracin 500 units per gram topical ointment; dextran, glycerin and hypromellose (GenTeal Tears) 0.1% and 0.2%, 0.3%, ophthalmic solution; mineral oil & white petrolatum (Refresh PM) 42.5%/57.3% ophthalmic ointment; sodium chloride (AYR saline) 0.9% intranasal spray. Table 2 is the emergency medical treatment medication pack, and all four medications in it contain publicly available expiry data from at least one source. Table 3 is the oral medication pack. There are five medications in it with no publicly available expiry data or did not meet inclusion criteria: fexofenadine (Allegra) 180 mg tablet [duplicate], benzonatate (Tessalon Perles) 100 mg softgel, hydrocodone/acetaminophen (Vicodin HP) 10 mg/300 mg tablets, meclizine (Antivert) 25 mg tablets, and modafinil (Provigil) 200 mg tablet [duplicate]. Table 4 is the topical & injectable medication pack. There are ten medications in it with no publicly available expiry data or did not meet inclusion criteria: benzocaine swabstick 20% topical swab, bacitracin 500 units per gram topical ointment [duplicate], ciprofloxacin/dexamethasone (Ciprodex) 0.3%/0.1% ophthalmic drops, erythromycin 0.5% ophthalmic ointment, cromolyn (Nasalcrom) 5.2 mg intranasal spray, diphenhydramine (Benadryl) 50 mg per mL injection, sterile water for injection, fluorescein strip 1 mg ophthalmic strips, desoximetasone (Topicort) 0.05% topical cream/gel, and cyanoacrylate (Dermabond) 0.5 mL topical swab. Table 5 is the vascular contingency medication pack, and there is one medication in it with no publicly available expiry data or did not meet inclusion criteria: sterile water for reconstitution.

**Table 1:** Shelf-life data for the convenience medication pack in months

ISS medication	US	UK/EU	NZ	AU
Antibiotic				
* Bacitracin 500* *u/gm, 0.9 unit dose pack*	N/A	N/A	N/A	N/A
Antidiarrheal				
Loperamide (Imodium) 2 mg Tablet	N/A	36–60	60	36
Antihistamine				
* Fexofenadine (Allegra) 180* *mg Tablet*	18–30	36	36	36
Loratadine (Claritin) 10 mg Tablet	N/A	36	24–36	24–36
Olopatadine (Pataday) 0.2%, 2.5 mL bottle	24	*24–36	*24–36	*24
Decongestant				
Oxymetazoline (Afrin) 0.05%, 15 mL bottle	N/A	36	30	30
Pseudoephedrine (Sudafed 12 hour) 120 mg Tablet	N/A	36	*36	36
Cough Relief				
Dextromethorphan (Robitussin) 15 mg Capsule	N/A	N/A	36	N/A
Lubricant				
Carboxymethylcellulose Sodium (Refresh Plus) 0.5%, 0.4 mL unit dose pack	N/A	18	N/A	N/A
Dextran, Glycerin and Hypromellose (GenTeal Tears) 0.1% and 0.2%, 0.3%, 15 mL bottle	N/A	N/A	N/A	N/A
Mineral Oil and White Petrolatum (Refresh PM) 42.5%; 57.3%, 3.5 gm tube	N/A	N/A	N/A	N/A
Sodium Chloride (AYR Saline) 0.9%, 22 mL bottle	N/A	N/A	N/A	N/A
Pain Reliever				
Acetaminophen (Tylenol) 325 mg Tablet	N/A	*36	*24–60	*18–48
Aspirin 325 mg Tablet	N/A	*36	*60	*24
Ibuprofen (Motrin) 400 mg Tablet	N/A	24–36	24–36	24–36
Sleep				
Melatonin 3 mg Tablet	N/A	18–48	36	24
Zaleplon (Sonata) 10 mg Bottle	N/A	*36	N/A	N/A
Zolpidem (Ambien) 5 mg Tablet	N/A	36	N/A	24
Steroid				
Hydrocortisone 1%, 0.9 gm unit dose pack	N/A	36–60	24–60	24–36
Mometasone (Nasonex) 50 mcg, 17 gm Bottle	N/A	24	36	36
Stimulant				
Caffeine (Vivarin) 200 mg Tablet	N/A	N/A	*36–48	*24–36
* Modafinil (Provigil) 200* *mg Tablet*	N/A	48	*24–60	*24–36
Stool Softener				
Bisacodyl (Dulcolax) 5 mg Tablet	N/A	24–36	36	24–36

Expiration data points are presented in months. Medication(s) in *italics* exist as a duplicate drug. Shelf-lives with an asterisk (*) represent data obtained from drug products of a differing strength than the ISS formulary. A “drug class” is presented with each group of medications, as reported in the ISS formulary.

**Table 2:** Shelf-life data for the emergency medical treatment medication pack in months

ISS medication	US	UK/EU	NZ	AU
Advanced life support (ALS) medications				
Epinephrine 0.1 mg/mL, 10 mL syringe	N/A	12–24	18	12–18
Overdose treatment				
Flumazenil (Romazicon) 0.1 mg/mL, 10 mL vial	N/A	36	24–60	24–60
Naloxone (Narcan) 1 mg/mL, 2 mL syringe	36	36	*24–36	*24–36
Severe allergic reaction				
Epinephrine (EpiPen) 0.3 mg/0.3 mL autoinjector	N/A	15–24	24	18–24

Expiration data points are presented in months. Shelf-lives with an asterisk (*) represent data obtained from drug products of a differing strength than the ISS formulary. A “drug class” is presented with each group of medications, as reported in the ISS formulary.

**Table 3:** Shelf-life data for the oral medication pack in months

ISS medication	US	UK/EU	NZ	AU
Altitude sickness				
Acetazolamide (Diamox) 250 mg Tablet	N/A	48	24–36	24
Antibiotic				
Amoxicillin (Amoxil) 500 mg Capsule	N/A	24–48	24	24–36
Azithromycin (Zithromax) 250 mg Tablet	N/A	48–60	36	36
Clindamycin (Cleocin) 300 mg Capsule	N/A	36	*60	*36–48
Doxycycline (Vibramycin) 100 mg Capsule	N/A	24–60	24	*24
Levofloxacin (Levaquin) 500 mg Tablet	N/A	36	N/A	N/A
Metronidazole (Flagyl) 500 mg Tablet	N/A	36	*36	*36
Sulfamethoxazole/Trimethoprim (Bactrim DS) 800/160 mg Tablet	N/A	60	*36	60
Antifungal				
Fluconazole (Diflucan) 150 mg Tablet	N/A	60	60	60
Antihistamine				
Diphenhydramine (Benadryl) 25 mg Capsule	N/A	24–48	*36	*36
* Fexofenadine (Allegra) 180* *mg Tablet*	18–30	36	36	36
Antiseizure				
Levetiracetam (Keppra) 500 mg Tablet	N/A	24–48	17–36	30–36
Antiviral				
Valacyclovir (Valtrex) 1 g Tablet	N/A	*12–36	36	24
Behavioral Health				
Aripiprazole (Abilify) 5 mg Tablet	N/A	24–48	24–36	36
Lorazepam (Ativan) 1 mg Tablet	N/A	24	20	20–24
Sertraline (Zoloft) 50 mg Tablet	N/A	24–60	48–60	36–60
Venlafaxine (Effexor XR) 75 mg Tablet	N/A	24–36	36	36
Cardiac				
Lisinopril (Zestril) 10 mg Tablet	N/A	24–48	36	24–60
Metoprolol XL (Toprol XL) 50 mg Tablet	N/A	N/A	36	24
Nitroglycerin (Nitrostat) 0.4 mg Tablet	N/A	*24	N/A	*24
Decongestant				
Pseudoephedrine (Sudafed) 30 mg Tablet	N/A	*36	*36	*24–48
Cough Relief				
Benzonatate (Tessalon Perles) 100 mg Softgel	N/A	N/A	N/A	N/A
Hormone				
Medroxyprogesterone (Provera) 10 mg Tablet	N/A	60	60	36–60
Norethindrone and Ethinyl Estradiol (Ortho Novum) 1 mg and 0.035 mg dose pack (21)	N/A	60	36	*36
Pain Reliever				
Hydrocodone/Acetaminophen (Vicodin HP) 10 mg/300 mg Tablet	N/A	N/A	N/A	N/A
Space Motion Sickness/Anti-Nausea				
Meclizine (Antivert) 25 mg Tablet	N/A	N/A	N/A	N/A
Ondansteron (Zofran ODT) 8 mg Tablet	N/A	36–60	24–36	24–36
Promethazine (Phenergan) 25 mg Tablet	24–60	36	24–60	24–60
Steroid				
Methylprednisolone (Medrol) 4 mg dose pack (21)	N/A	36–60	18–60	N/A
Prednisone (Deltasone) 10 mg Tablet	N/A	N/A	24	N/A
Stimulant				
* Modafinil (Provigil) 200* *mg Tablet*	N/A	48	*24–60	*24–36
Stomach				
Bismuth Subsalicylate (Pepto Bismol) 262 mg Chewable	N/A	*36	N/A	N/A
Omeprazole (Prilosec) 20 mg Capsule	N/A	12–36	36	18–36
Famotidine (Pepcid) 20 mg Tablet	30	36	36	12–36
Urinary				
Tamsulosin (Flomax) 0.4 mg Capsule	N/A	36	24	36
Nitrofurantoin Monohydrate (Macrobid) 100 mg Capsule	N/A	24–48	24–60	24–60

Expiration data points are presented in months. Medication(s) in *italics* exist as a duplicate or were excluded from final analysis. Shelf-lives with an asterisk (*) represent data obtained from drug products of a differing strength than the ISS formulary. A “drug class” is presented with each group of medications, as reported in the ISS formulary.

**Table 4:** Shelf-life data for the topical & injectable medication pack in months

ISS medication	US	UK/EU	NZ	AU
Anesthetic				
Dental				
Benzocaine Swabstick 20%, 0.15 mL Swab	N/A	N/A	N/A	N/A
Eye				
Tetracaine 0.5%, 15 mL bottle	N/A	18	30	30
Local				
Lidocaine and Epinephrine (Xylocaine and Epinephrine) 2% with 1:100,000 Epi, 20 mL multi-dose vial	N/A	*24	24	18
Lidocaine (Xylocaine) 1%, 10 mL multi-dose vial	N/A	24–48	18–48	14–36
Urinary				
Lidocaine Jelly 2%, 30 mL Tube	N/A	N/A	24	24
Antibiotic				
* Bacitracin 500* *u/gm, 28* *gm Tube*	N/A	N/A	N/A	N/A
Ceftriaxone (Rocephin) 1 g single-dose vial	N/A	24–36	36	24–36
Ciprofloxacin/Dexamethasone (Ciprodex) 0.3%/0.1%, 7.5 mL bottle	N/A	N/A	N/A	N/A
Ertapenem (Invanz) 1 g single-dose vial	N/A	24	24	24
Erythromycin 0.5%, 3.5 g Tube	N/A	N/A	N/A	N/A
Moxifloxacin (Vigamox) 0.5%, 3 mL Bottle	N/A	24–36	N/A	N/A
Mupirocin (Bactroban) 2%, 22 g Tube	N/A	18–24	24	18–36
Tobramycin/Dexamethasone (Tobradex) 0.3%/0.1%, 10 mL Bottle	N/A	24	24	N/A
Antifungal				
Clotrimazole (Lotrimin) 1%, 30 g Tube	N/A	24–36	36	12–48
Antihistamine				
Cromolyn (Nasalcrom) 5.2 mg, 26 mL Bottle	N/A	N/A	N/A	N/A
Diphenhydramine (Benadryl) 50 mg/mL, 1 mL single-dose vial	N/A	N/A	N/A	N/A
Behavioral health				
Diazepam (Valium) 5 mg/mL, 2 mL syringe	N/A	24–36	36	36
Ziprasidone (Geodon) Medication Kit				
Ziprasidone (Geodon) 20 mg/mL, 1.2 mL single-dose vial	N/A	N/A	N/A	24
* Sterile Water for Injection, 10* *mL single-dose vial*	N/A	N/A	N/A	N/A
Breathing				
Albuterol (Proventil HFA) 90 mcg, 6.7 g Inhaler	N/A	*24–36	*24	*24
Fluticasone (Flovent HFA) 220 mcg, 12 g Inhaler	N/A	*24–36	*24–36	*24–36
Salmeterol (Serevent) 50 mcg Inhaler	N/A	18–24	24	24
Dental				
Eugenol 1 mL Oral Syringe	N/A	36	N/A	N/A
Earwax Removal				
Carbamide Peroxide (Debrox) 6.5%, 15 mL Bottle	N/A	*24	24	18
Eye				
Cyclopentolate (Cyclogyl) 1%, 15 mL Bottle	N/A	15–24	24–30	18–30
Fluorescein Strip 1 mg Strip	N/A	N/A	N/A	N/A
Tropicamide (Mydriacyl) 1%, 15 mL Bottle	N/A	15–36	30–36	30–36
Intubation				
Glycopyrrolate (Robinul) 0.2 mg/mL, 2 mL single-dose vial	N/A	18–24	24–36	24–36
Ketamine (Ketalar) 50 mg/mL, 10 mL multi-dose vial	N/A	30–60	*36	36
Pain reliever				
Hydromorphone (Dilaudid) 2 mg/mL, 1 mL Syringe	N/A	36	N/A	32–36
Ketorolac (Toradol) 30 mg/mL, 2 mL single-dose vial	24	24–36	N/A	24
Space Motion Sickness/Anti-Nausea				
Promethazine (Phenergan) 25 mg/mL, 1 mL single-dose vial	24–60	36	36	36
Steroid				
Dexamethasone (Decadron) 10 mg/mL, 1 mL single-dose vial	N/A	*18–24	*18–24	*18–24
Flucinonide (Lidex) 0.05%, 30 g Tube	N/A	36	N/A	N/A
Desoximetasone (Topicort) 0.05%, 60 g Tube	N/A	N/A	N/A	N/A
Wound repair				
Chemical Cautery				
Silver Nitrate 75%; 25% Each	60	N/A	N/A	N/A
Skin Adhesive				
Cyanoacrylate (Dermabond) 0.7 mL Swab	N/A	N/A	N/A	N/A

Expiration data points are presented in months. Medication(s) in *italics* exist as a duplicate or were excluded from final analysis. Shelf-lives with an asterisk (*) represent data obtained from drug products of a differing strength than the ISS formulary. A “drug class” is presented with each group of medications, as reported in the ISS formulary.

**Table 5:** Shelf-life data for the vascular contingency medication pack in months

ISS medication	US	UK/EU	NZ	AU
Anticoagulants				
Aspirin 81 mg tablet	N/A	*21–36	*24	*18–36
Apixaban (Eliquis) 2.5 mg tablet	N/A	24–48	36	36
Apixaban (Eliquis) 5 mg tablet	N/A	24–48	36	36
Atorvastatin 80 mg tablet	N/A	24–36	24–36	16–48
Clopidogrel (Plavix) 75 mg tablet	N/A	24–36	36	18–36
Enoxaparin (Lovenox) 300 mg/3 mL multi-dose vial	N/A	24–36	36	24–36
Sodium Chloride flush (normal saline) 0.9%, 5 mL syringe	N/A	9–36	15–24	15–60
Reversal agents				
Protamine Sulfate 10 mg/mL, 25 mL single-dose vial	N/A	48	36	48
Prothrombin Complex Concentrate (KCentra) 1000units single-dose vial	36	36	N/A	36
* Sterile Water for Reconstitution, 50* *mL vial*	N/A	N/A	N/A	N/A
Antihypertensive				
Metoprolol (Lopressor) 50 mg tablet	N/A	24	*36	*24–36

Expiration data points are presented in months. Medication(s) in *italics* were excluded from final analysis. Shelf-lives with an asterisk (*) represent data obtained from drug products of a differing strength than the ISS formulary. A “drug class” is presented with each group of medications, as reported in the ISS formulary.

## Discussion

**Fig. 1:**
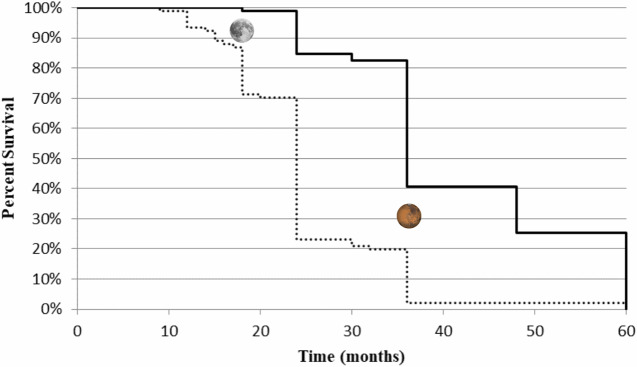
Kaplan-Meier curve of the ISS formulary shelf-lives. Kaplan-Meier curve of the cumulative number of medications in the 2023 ISS formulary with publicly available shelf-lives (*n* = 91) over five years. The maximum estimated shelf-life across all sources for each medication is visualized with the solid line, and the minimum with the dashed line to predict survival. The image of the moon at 18 months depicts the approximate duration of a mission to and from the moon, while the image of mars at 36 months depicts the approximate duration of a mission to and from mars.

Much of the expiry data in this publicly available repository are presented as a range due to the varying shelf-lives between manufacturers of the same drug product, such as brand versus generic products. Additionally, the shelf-lives represent the expiration for each drug product in its original packaging, and thus may be shorter if repackaged such as with the medications in the ISS^5,8–11,18,20,21^. The lack of expiration data available for 14% (*n* = 15) of the 2023 ISS formulary presents the risk of therapeutic failure for humans in extended space missions. A crewed mission to and from Mars, with ample time on the planet’s surface, may take approximately 24 to 36 months^22^. Using the maximum labeled shelf-life across all sources for each medication, we found that 14 medications will expire by 24 months: one ophthalmic lubricant, one advanced life support medication, one anaphylaxis treatment medication, one benzodiazepine, one antiangina medication, two corticosteroids, one local anesthetic, one topical urinary jelly, two antibiotics, one antipsychotic, one inhaler, and one ear wax removal medication. Furthermore, over half (*n* = 54) of the entire 2023 ISS formulary will expire by 36 months; this is demonstrated in the Kaplan-Meier survival curve in Fig. 1. The earliest medication expiration begins at 18 months, presenting a minimal risk for an extended mission to the moon: one ophthalmic lubricant. Ultimately, these findings amplify the risk of therapeutic failure when treating an adverse health condition in an extended space expedition.

Due to pharmaceutical company proprietary concerns, it is difficult to obtain US expiry data from the FDA^23^. This required us to consult drug databases in online repositories from other countries with similar drug stability standards as the US^24–26^. However, the ISS formulary shelf-lives may differ from the shelf-lives of medications manufactured in other countries due to differences in excipients, packaging, and re-test standards^9^. For example, we found promethazine tablets possess a shelf-life of up to 60 months from manufacturers in NZ and AU, but a shelf-life of 36 months in the UK/EU. Importation of medications and collaboration with other countries may therefore be a future consideration. Another logistical consideration for supplying a self-sufficient pharmacy in space pertains to pharmaceutical supply chains. The pharmaceutical industry is a complex landscape with many factors affecting the dynamics of its supply chains, often resulting in delays to the distribution of a drug product. The stepwise process from primary manufacturing of the API to the distribution of the final drug product to wholesalers and retailers may take up to 300 days^27^. This is due to delays from quality control, factory equipment and cleaning, potential coordination with contractors, and transportation of supplies, among others^27^. By the time NASA receives and packages their drug products for spaceflight, the products may have already lost a year of their shelf-life. Therefore, a product’s functional shelf-life can be even shorter than the labeled shelf-life. In rare scenarios, drug shortages occur, which can impact NASA mission planning. Management of drug demand at a manufacturer level is based on historical data and market intelligence in each geographic location^27^. In the context of supplying medications in space, this bottleneck effect can be anticipated from market trends, but ultimately there is little that can be done on the consumer level. The use of logistics data and trends through data mining may be useful for NASA and its counterparts.

Based on comments within the ISS formulary, the two sterile water diluents we excluded are intended for use with alternate medications: ziprasidone (Geodon) 20 mg per mL injection and prothrombin complex concentrate (KCentra) 1000 units. Moreover, packaging information from manufacturers of both of these products describe a diluent included with their drug product. For this reason, we assumed that these diluents likely have the same shelf-life as the actual drug product it is packaged with. However, we found that the labeled shelf-life of sterile water ranges from 18 to 60 months across all sources, depending on the container type (i.e. bags, vials, or ampoules) and size.

We found data for benzocaine 20% gel but did not include this in the final analysis due to a likely different formulation than the benzocaine 20% swabstick used in the ISS. The shelf-life of benzocaine gel is 36 months from EU sources. Similarly, a 500 mg coated granule formulation of levetiracetam was found from UK/EU sources but was not included in the final analysis. The shelf-life of these coated granules in sachets is 60 months. Cromolyn (Nasalcrom) 5.2 mg intranasal spray exists in the US as an OTC product, but is unavailable in this dosage form in the UK/EU, NZ, and AU; therefore, no data was found for this product. While Zaleplon (Sonata) was discontinued for marketing and distribution in the EU in 2015, the shelf life data used in our analysis for this medication was from an EU source^28^.

Blue et al. explored the challenges and current understanding of supplying a pharmacy for space exploration in 2019, but did not include a list of every medication in the ISS and their shelf-life^9^. However, Blue et al. did provide preliminary information from emc of the shelf-lives of 42 medications aboard the ISS, two of which are the same drug in different dosage forms (Diphenhydramine tablet and injectable and promethazine tablet and injectable)^9^. Aside from four of the 42 medications explored in Blue et al. (levofloxacin 500 mg tablet, modafinil 200 mg tablet, tamsulosin 0.4 mg capsule, and diphenhydramine 50 mg per mL), the shelf-life data obtained in our study align with previously reported emc-obtained data. Therefore, our study is more comprehensive due to inclusion of all medications routinely stored on board the ISS as of 2023. Furthermore, our study also includes data from sources outside of the US and UK/EU, such as NZ and AU. However, there is one medication included in the Blue et al. analysis which was not listed in the 2023 ISS formulary: triamcinolone cream^9^. This is likely due to an update in the ISS formulary since publication. Triamcinolone cream is not included in our analysis, but the reported shelf-life from Blue et al. is 36 months^9^. Additionally, sodium chloride (normal saline) is included in the Blue et al. analysis, but the exact dosage form is not specified; the reported shelf-life was 36 months^9^.

While drug products naturally degrade over time, studies have alluded to degradation at an accelerated rate in space^29–35^. Du et al. found 25 of 36 medicines stored for 880 days (~2.5 years) on the ISS did not meet USP requirements for API strength, compared to the control group of 17 of 36 medicines at the same time point^29^. This is the only published study comparing the stability of medications after space flight to a control group on Earth after a set period of time^11,29^. However, medications stored on board the ISS are often repackaged in different containers than the stability studies conducted on Earth^5,8–11,18,20^. While interplanetary radiation may play a role in medication degradation, recent evidence proposes that drug repackaging prior to transportation to space may drive the accelerated degradation of drug stability^11^. Therefore, it should be noted the data presented in our study is merely an estimate of the terrestrial shelf-lives of the medications listed, and does not represent the shelf-lives of these medications in space. Regardless, our data contain valuable evidence for future investigations of space exploration medical capability^5^.

The ISS formulary is frequently updated based on growing evidence of medication use in microgravity environments. For example, a publicly available document containing the 2016 ISS formulary includes 95 medications, but a 2017 NASA evidence report cites 107 medications in the ISS formulary^23^. This 2017 report also acknowledges information about the expiration dates of the ISS formulary medications based on FDA standards, suggesting that NASA may have a baseline knowledge of the proposed terrestrial shelf-lives of their medications^23^. It is unclear as to whether NASA utilizes brand or generic medications in the medication kits aboard the ISS; this may result in varying shelf-lives. In addition, this study may not include medications that might be used for commercial astronauts and space “tourists.”

In our results tables, we indicate with an asterisk whether a data point was collected for a drug of a differing strength than what is contained in the ISS formulary. This is because some pharmaceutical companies from varying countries do not always manufacture the same drug strength as products in the US. For example, data for aspirin 81 mg tablets could not be found in the UK/EU, NZ, or AU, but these countries contained data for aspirin 75 mg or 100 mg tablets instead.

Some of the expiration data points of the drug products collected in our analysis exist in different dosage forms, containers, or packaging compared to the ISS. For example, data for capsules, caplets, softgels, and tablets were obtained synonymously, and are stored in varying containers depending on the manufacturer, such as high density polyethylene (HDPE) bottles or polyvinyl chloride (PVC) blister packs. Additionally, data for vials, syringes, and ampoules for injection were obtained synonymously, but it is unlikely that ampoules may be used safely in space due to debris and inability to draw up the liquid into a syringe in a microgravity environment. This presents a vast limitation in some of the data presented in this study, particularly for the injectable products. One such situation in which our expiration data may not accurately represent the shelf-life of the drug product contained in the ISS is with sodium chloride flush (normal saline) 0.9%. Based on our inclusion criteria, we collected shelf-life data for sodium chloride vials, ampoules, and bags; data points for sodium chloride syringes could not be found. The shelf-life for this product ranged from 15 to 60 months. Similarly, sterile water is supplied in many different containers. As stated earlier, we chose to exclude both sterile water products in our analysis because comments in the ISS formulary describe these products as diluents for both ziprasidone and prothrombin complex concentrate. However, we recognize that many of the injectable products, such as the antibiotics, likely exist as a lyophilized powder and require reconstitution prior to use. Therefore, it is assumed that other diluents may exist in the ISS. The range of shelf-lives that we found for sterile water are 18 to 60 months, depending on the packaging and size.

As previously mentioned, our study solely focuses on the terrestrial expiry data of drugs in their original storage conditions and does not take into account the risk of accelerated expiration of pharmaceuticals stored in space for extended periods of time, such as due to space radiation^11^. Lastly, the authors acknowledge this analysis is based on publicly available data through August 2023 and is subject to change based on future stability studies surrounding the aforementioned drug products.

There is still much research to be conducted regarding the safe and efficacious use of medications for long-duration space flight missions. Several studies have explored the pharmacokinetic and pharmacodynamic (PK/PD) properties of medications in microgravity environments^36–40^. In-vivo PK/PD studies of commonly used ISS medications may guide future clinical guidelines for best practice dosing regimens in humans in space. Limited data is available regarding in-depth tracking of medication usage amongst humans during spaceflight^6,7^. One study utilized a novel iOS application to collect medication use data from volunteer astronauts during spaceflight, but usability was the primary criticism^7^. Advancements in software-based dose-tracking applications may assist with formulary selection, understanding medication tolerance, and pharmacovigilance in space. Pharmacogenomics, described as the impact of genetic variation on drug response, has been suggested to support pharmacological treatment in astronauts^41^. Stingl et al. found approximately a third of medications routinely stored in the ISS may be affected by genetic polymorphisms^41^. A deeper analysis of these medications and the extent of their effect on medication safety and effectiveness may provoke consideration of pharmacogenetic testing prior to spaceflight as well as personalized dosing for astronauts.

The US Shelf-Life Extension Program (SLEP) investigates terrestrial expiration date extensions of select drug products for emergency preparedness^42,43^. For example, select lot numbers of doxycycline hyclate 100 mg capsules were approved for an expiration extension of over four years beyond the manufacturer’s originally labeled expiry date for an anthrax emergency preparedness response^44^. Other studies have demonstrated that many medications remain stable beyond their labeled expiration dates when stored in their original packaging^45–50^. Future expiration extension studies may provide a deeper understanding of under what conditions the expiration dates of medications can be extended for use in deep-space missions. Some pharmaceutical companies, such as Merck and Eli Lilly and Company, have begun exploring alterations in physical and chemical properties of medications in microgravity environments^51,52^. This may eventually lead to onboard manufacturing of space medicines such as through 3-D printing or on-demand manufacturing via miniaturized portable systems^53–56^. Although new stability guidance would likely be established for a pharmaceutical product manufactured in space, this concept may provide a solution for drug stability during EIMO.

Pharmaceuticals will likely be the cornerstone of maintaining the health and performance of humans participating in exploration space missions. There is a gap in public knowledge in the prospective shelf-lives of the medications contained in the ISS formulary. It is imperative to know and understand these pharmacologic parameters in order to supply a safe and effective *astropharmacy*. The range of shelf-lives obtained from various international sources in this study provide insight for supplying a pharmacy for exploration spaceflight and may also provide foundational evidence for a deeper analysis of expiration extension studies. A lack of expiration data for 14% of the ISS medications shows the necessity for collaboration and transparency between pharmaceutical suppliers and spaceflight operators. More than half of the entire ISS formulary will expire before 36 months, the approximate duration of an extended mission to and from Mars. Instability of pharmaceutical agents increases the risk of therapeutic failure, and may complicate mission planning. NASA and its international partners may have the capability to design a self-sufficient pharmacy in space or on other terrestrial bodies through strategically planned research efforts to address the gaps in knowledge presented in this analysis. Ultimately, those responsible for the health of spaceflight crews will have to find ways to extend the expiration of medications to the complete mission duration or accept the elevated risk associated with administration of an expired medication.

## Methods

### Obtaining the shelf-life data

The medications in each medication kit within the ISS formulary were cross-referenced with each drug’s respective, publicly available US new drug application (NDA) and monograph, as well as the 2023 American Hospital Formulary Service (AHFS) Drug Information compendium to identify US shelf-life data. Then, we consulted four drug databases in online repositories from other countries to compare and explore expiry data. We used the United States Pharmacopeia (USP) Dictionary to identify international nomenclature equivalents of certain medications. The Electronic Medicines Compendium (emc) and Medthority were used in accordance with the European Medicines Agency (EMA) and Medicines and Healthcare products Regulatory Agency (MHRA) to obtain United Kingdom (UK) and European (EU) shelf-life data; MedSafe was used to obtain New Zealand (NZ) shelf-life data; Australian Register of Therapeutic Goods (ARTG) was used to obtain Australian (AU) shelf-life data. Shelf-life data from other publicly available sources were assessed for credibility utilizing a Stanford Credibility Tool, in which the sources were assessed for expertise, trustworthiness, reproducibility, and quality and given a score of 0–4.

### Analysis of the shelf-life data

**Fig. 2:**
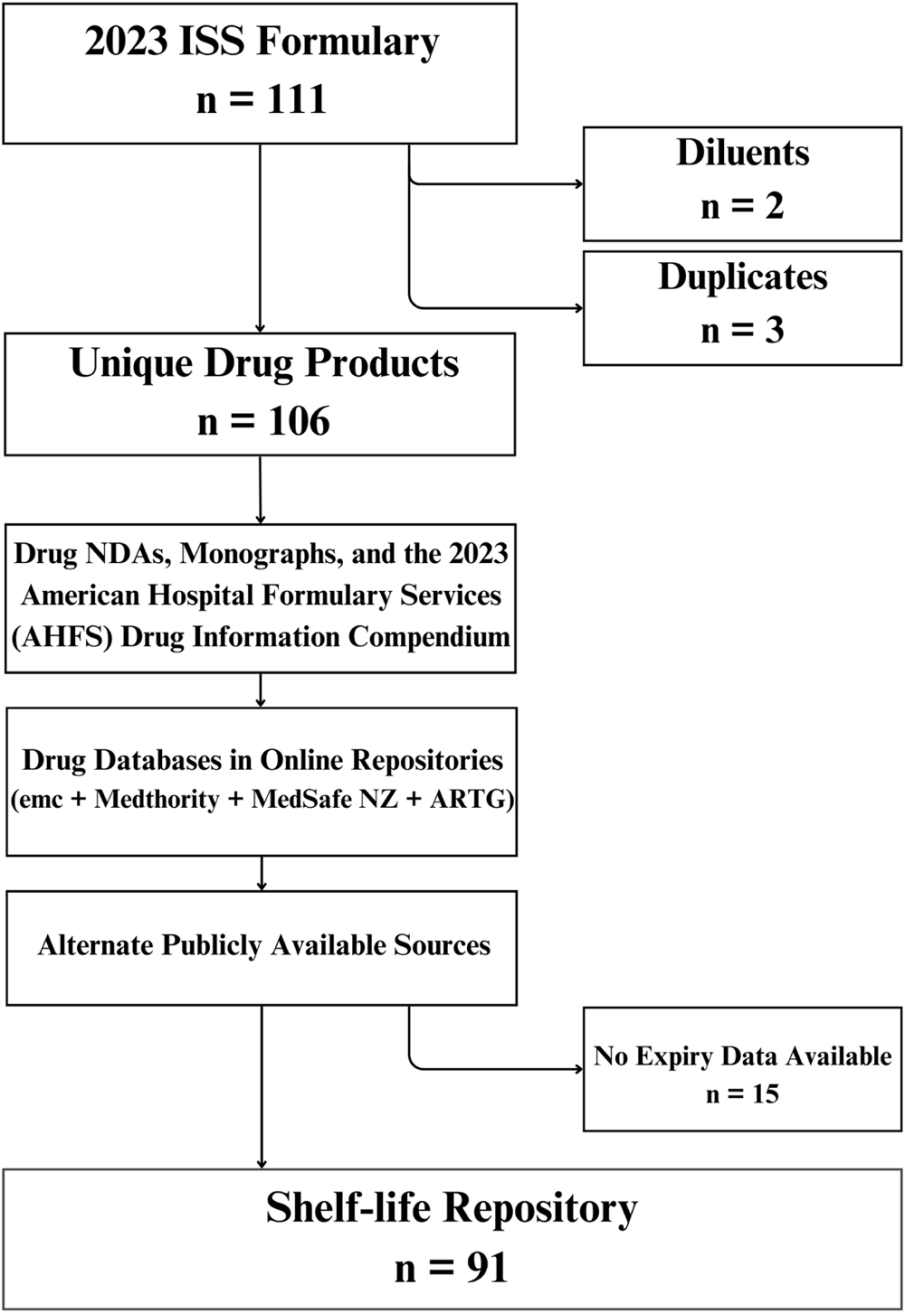
Stepwise process to collect shelf-life data. A visual schema of the stepwise process undertaken to scrutinize the ISS formulary, starting with the inclusion and exclusion of drug products.

Expiration data points were scrutinized descriptively and stored in Microsoft Excel. The data were presented in tables indicating each medication in the ISS formulary with a range of estimated shelf-lives from each international source. Figure 2 provides a simplified pipeline of the project methodology. For analysis, both the minimum and maximum labeled shelf-life of each medication across all sources was scrutinized. For example, if a medication contains a shelf-life of 24–48 months in the UK/EU but 36 months in NZ and 60 months in AU, the minimum shelf-life was considered 24 months and the maximum 60 months. This method was used to create two Kaplan-Meier survival curves - one using the minimum shelf-lives and one using the maximum shelf-lives. The Kaplan-Meier survival curves were created manually using a Microsoft Excel Kaplan-Meier add-in.

### Inclusion

Medications included in our analysis are: (1) over-the-counter (OTC) and prescription products; (2) brand and generic products; (3) products that are of a similar dosage form and contain the same API, route of administration, and strength as the ISS formulary. If a shelf life for a certain drug strength was unavailable, the next closest drug strength was obtained, as indicated with an asterisk in our results.

### Exclusion

Medications were excluded from our analysis if: (1) a product has a different route of administration than the respective ISS formulary product; (2) exists in a combination product with a differing API not listed on the ISS formulary; (3) is no longer available for marketing or distribution in the US. Additionally, specific medications were excluded on a case-by-case basis depending on indication, formulation, or purpose compared to the ISS formulary.

## Data Availability

All data supporting this research are publicly available, and may be found in previous publications cited within the text.

## Code availability

The underlying code for this study is available in GitHub and can be accessed via this link: https://github.com/lukashalim/ExcelSurvival.

## References

[1] Lewis R. Human System Risk Board. *National Aeronautics and Space Administration (NASA)*. (2022).

[2] Goldin D. S. (1999, Oct 25). Planning an Infrastructure for Astronaut Health Care [Speech]. US National Academy of Sciences, Washington (DC). As Published in Safe Passage: Astronaut Care for Exploration Missions (2001).

[3] Sands K. NASA Manages Astronaut Health with Effective Diagnostics Research. *National Aeronautics and Space Administration (NASA)* (2022).

[4] Lewis R. Ineffective or Toxic Medication Effects. *National Aeronautics and Space Administration (NASA)* (2022).

[5] Wotring V. E. Evidence Report: Risk of Therapeutic Failure Due to Ineffectiveness of Medication. *National Aeronautics and Space Administration (NASA)* (2011).

[6] Wotring, V. E. Medication use by U.S. crewmembers on the International Space Station. *FASEB J.* **29**, 4417–4423 (2015). 26187345 10.1096/fj.14-264838

[7] Wotring, V. E. & Smith, L. K. Dose tracker application for collecting medication use data from International Space Station Crew. *Aerosp. Med. Hum. Perform.* **91**, 41–45 (2020). 31852573 10.3357/AMHP.5392.2020

[8] Tran, Q. D. et al. Space medicines for space health. *ACS Med Chem. Lett.* **13**, 1231–1247 (2022). 35978686 10.1021/acsmedchemlett.1c00681 PMC9377000

[9] Blue, R. S. et al. Supplying a pharmacy for NASA exploration spaceflight: challenges and current understanding. *NPJ Microgravity* **5**, 14 (2019). 31231676 10.1038/s41526-019-0075-2 PMC6565689

[10] Nimon J. Preparing to Stock the Medicine Cabinet for Long-Duration Missions. *National Aeronautics and Space Administration (NASA)* (2011).

[11] Reichard, J. F. et al. The effect of long-term spaceflight on drug potency and the risk of medication failure. *NPJ Microgravity* **9**, 35 (2023). 37147378 10.1038/s41526-023-00271-6 PMC10163248

[12] U.S. Food and Drug Administration (FDA). Q1A(R2) Stability Testing of New Drug Substances and Products. *U.S. Department of Health and Human Services* (2003).

[13] Mehta, P. & Bhayani, D. Impact of space environment on stability of medicines: challenges and prospects. *J. Pharm. Biomed. Anal.* **136**, 111–119 (2017). 28068518 10.1016/j.jpba.2016.12.040

[14] Carstensen, J. & Rhodes C. *Drug Stability: Principles and Practices*, 3rd edn, CRC Press, New York, NY, (2000).

[15] Capen, R. et al. On the shelf life of pharmaceutical products. *AAPS PharmSciTech* **13**, 911–918 (2012). 22729779 10.1208/s12249-012-9815-2 PMC3429690

[16] USP. <7> Labeling, General Chapter. United States Pharmacopeia 43, 6435 (2019).

[17] U.S. Food and Drug Administration (FDA). Q3A Impurities in New Drug Substances. *U.S. Department of Health and Human Services* (2008).

[18] Wotring V. *Space Pharmacology*, Springer: International Space University, New York, NY, (2012).

[19] Nguyen, V. Pharmacists out of this world. *CPJ* **151**, 366–367 (2018). 30559909 10.1177/1715163518804766 PMC6293404

[20] Daniels, V. & Bayuse, T. Risk of ineffective or toxic medications during long-duration exploration spaceflight. Oral Presentation, NASA Human Systems Risk Board (2018).

[21] U.S. Food and Drug Administration (FDA). Repackaging of Certain Human Drug Products by Pharmacies and Outsourcing Facilities. *U.S. Department of Health and Human Services* (2017).

[22] Tillman N. T. & Dobrijevic D. How long does it take to get to Mars? *Space* (2023).

[23] Antonsen, E. et al. Evidence report: risk of adverse health outcomes and decrements in performance due to in-flight medical conditions. National Aeronautics and Space Administration, Houston, Tx, USA (2017).

[24] European Medicines Agency (EMA). ICH Topic Q1A(R2) Stability Testing of new Drug Substances and Products. *European Union* (2006).

[25] Therapeutic Goods Administration (TGA). Stability testing for prescription medicines. *Australian Government: Department of Health* (2017).

[26] Medsafe: New Zealand Medicines and Medical Devices Safety Authority. New Zealand Regulatory Guidelines for Medicines. *Ministry of Health* (2014).

[27] Shah, N. Pharmaceutical supply chains: key issues and strategies for optimisation. *Comput. Chem. Eng.* **28**, 929–941 (2004). 10.1016/j.compchemeng.2003.09.022

[28] European Medicines Agency (EMA). Public Statement: Sonata. *European Union* (2015).

[29] Du, B. et al. Evaluation of physical and chemical changes in pharmaceuticals flown on space missions. *AAPS J.* **13**, 299–308 (2011). 21479701 10.1208/s12248-011-9270-0 PMC3085701

[30] Wu, L. & Chow D. Degradation Analysis of Medications from ISS Using LC-MS/MS Assays: NSBRI RFA 15-01 First Award Fellowship, Final Report. Tech. Rep., National Space Biomedical Research Institute (2016).

[31] Cory W. C., James V., Lamas A., Mangiaracina K., Moon J. Analysis of degradation of pharmaceuticals stored on the international space station. *National Aeronautics and Space Administration (NASA)*, 1–25 (2016).

[32] Cory W. C., James V., Mangiaracina K. Analysis of degradation of pharmaceuticals stored on the international space station - final report. N*ational Aeronautics and Space Administration (NASA)*, 1–19 (2017).

[33] Wotring, V. E. Chemical potency and degradation products of medications stored over 550 earth days at the international space station. *AAPS J.* **18**, 210–216 (2016). 26546565 10.1208/s12248-015-9834-5 PMC4706284

[34] Khan, M. & Wotring, V. E. FDA comprehensive stability evaluation of three medications. *National Aeronautics and Space Administration (NASA)*, 1–82 (2014).

[35] Chuong, M. C., Prasad, D., Leduc, B., Du, B. & Putcha, L. Stability of vitamin B complex in multivitamin and multimineral supplement tablets after space flight. *J. Pharm. Biomed. Anal.* **55**, 1197–1200 (2011). 21515013 10.1016/j.jpba.2011.03.030

[36] Cintron, N. M. et al. Inflight pharmacokinetics of acetaminophen in saliva. In: Bungo M. W., Bagian T. M., Bowman M. A. editors. NASA technical memorandum 58280. Results of the life sciences DSOs conducted aboard the space shuttle 1981-1986. Houston, TX: National Aeronautics and Space Administration, 19–23 (1987).

[37] Kovachevich, I. V. et al. Pharmacokinetics of acetaminophen administered in tablets and in capsules under long term space flight conditions. *Pharm. Chem. J.* **43**, 130–133 (2009). 10.1007/s11094-009-0255-6

[38] Cintron, N. M. et al. Inflight salivary pharmacokinetics of scopolamine and dextroamphetamine. In: Bungo M. W., Bagian T. M., Bowman M. A., et al., editors. NASA technical memorandum 58280. Results of the life sciences DSOs conducted aboard the space shuttle 1981-1986. Huston, TX: National Aeronautics and Space Administration, 25–29 (1987).

[39] Boyd J., Wang Z., Putcha L. Bioavailability of promethazine during spaceflight. Tech. Rep. NASA/TM-2009-01322, NASA Johnson Space Center (2009).

[40] Putcha, L. & Kovachevich, I. Physiologic alterations and pharmacokinetic changes during space flight. NASA Life Sciences Portal.

[41] Stingl, J. C. et al. Where Failure is Not an Option - Personalized Medicine in Astronauts. *PloS One* **10**, e0140764 (2015). 26489089 10.1371/journal.pone.0140764 PMC4619198

[42] Approaches to Drug Product Expiration Date Extensions: Shelf-Life Extension Program. *U.S. Food and Drug Administration (FDA)* (2023).

[43] Khan, A. R. et al. United States Food and Drug Administration and Department of Defense shelf-life extension program of pharmaceutical products: progress and promise. *J. Pharm. Sci.* **103**, 1331–1336 (2014). 24623105 10.1002/jps.23925

[44] Bumpus N. N. Update on Expiration Date Extensions of Certain Lots of Doxycycline Hyclate 100 mg Capsules Held in Strategic Stockpiles. *U.S. Food and Drug Administration (FDA)* (2023).

[45] Lyon, R., Taylor, J., Porter, D., Prasanna, H. & Hussain, A. Stability profiles of drug products extended beyond labeled expiration dates. *J. Pharm. Sci.* **96**, 1549–1560 (2006). 10.1002/jps.20636 16721796

[46] Matto, V. & Meos A. in *Microbial Pathogens and Strategies for Combating them: Science, Technology and Education*. (Formatex Research Center, Badajoz, Spain (ed. Méndez-Vilas, A.) 1721–1725 (2013).

[47] Cantrell, L., Suchard, J. R., Wu, A. & Gerona, R. R. Stability of active ingredients in long-expired prescription medications. *Arch. Intern Med.* **172**, 1685–1687 (2012). 23045150 10.1001/archinternmed.2012.4501

[48] Weir, W. B. et al. Expired epinephrine maintains chemical concentration and sterility. *Prehosp. Emerg. Care* **22**, 414–418 (2018). 29373043 10.1080/10903127.2017.1402109

[49] Cantrell, F. L. et al. Epinephrine concentrations in EpiPens after the expiration date. *Ann. Intern. Med.* **166**, 918–919 (2017). 28492859 10.7326/L16-0612

[50] Browne, E., Peeters, F., Priston, M. & Marquis, P. T. Expired drugs in the remote environment. *Wilderness Environ. Med.* **30**, 28–34 (2019). 30718138 10.1016/j.wem.2018.11.003

[51] Reichert, P. et al. Pembrolizumab microgravity crystallization experimentation. *NPJ Microgravity* **5**, 28 (2019). 31815178 10.1038/s41526-019-0090-3 PMC6889310

[52] National Aeronautics and Space Administration Science (NASA) in Short: Eli Lilly-Hard to Wet Surfaces.

[53] Prater, T. et al. 3D Printing in Zero G technology demonstration mission: Complete experimental results and summary of related material modeling efforts. *Int. J. Adv. Manuf. Technol.* **101**, 391–417 (2019). 32454552 10.1007/s00170-018-2827-7 PMC7243176

[54] Zhu, X. et al. 3D printing promotes the development of drugs. *Biomed. Pharmacother.* **131**, 110644 (2020). 32853908 10.1016/j.biopha.2020.110644

[55] Arnold, C. Who shrank the drug factory? Briefcase-sized labs could transform medicine. *Nature* **575**, 274–277 (2019). 31719703 10.1038/d41586-019-03455-x

[56] Adiga, R. et al. Point-of-care production of therapeutic proteins of good-manufacturing-practice quality. *Nat. Biomed. Eng.* **2**, 675–686 (2018). 31015674 10.1038/s41551-018-0259-1

